# Non Typhoidal Salmonella Pyonephrosis in an Asymptomatic Immunocompetent Patient

**DOI:** 10.1155/2019/4198275

**Published:** 2019-09-22

**Authors:** Seif Mokadem, Mohamed Ali Nouioui, Salma Kalai, Tarek Taktak, Houssem Mediouni, Ramzi Khiari, Samir Ghozzi

**Affiliations:** ^1^Department of Urology, Military Hospital of First Instruction of Tunis, Tunisia; ^2^Department of Microbiology, Military Hospital of First Instruction of Tunis, Tunisia

## Abstract

A 50-year-old man with no past medical history presented with 5 months history of right flank discomfort. Physical examination was unremarkable. CT-scan showed a large right renal pelvic calculi and upper pole hydronephrosis. He underwent open surgical procedure and we peroperatively discovered upper pole pyonephrosis. Bacteriological samples of pus grew group D Salmonella. We prescribed third generation cephalosporin for 14 days. The patient made a steady recovery. Non typhoidal salmonella (NTS) urinary tract infection (UTI) is extremely rare and usually associated with immunosuppressive chronic disease or genito urinary tract abnormalities. Pyonephrosis due to NTS have been reported twice. We report the first case of asymptomatic NTS pyonephrosis.

## 1. Introduction

Non typhoidal salmonella (NTS) is an enteroinvasive bacterium. Non typhoidal salmonellosis may have different clinical presentations [1]. Gastroenteritis is the most common form [1]. Urinary tract infection (UTI) due to NTS is extremely rare [2]. It is usually associated with immunosuppression or abnormalities of the genitourinary tract [1, 2]. The infection can be hematogenous or due to direct urethral invasion.

We present an unusual case of an incidental peroperative discovery of pyonephrosis due to NTS in an asymptomatic immunocompetent 50-year-old man.

## 2. Case Presentation

A 50-year-old man with no past medical history, presented with 5 months history of recurrent right flank discomfort. He denied any hematuria, passage of calculi, fever, chills, vomiting, diarrhea, constipation, or abdominal pain. He was not on any medication.

On examination, he was afebrile. Abdominal examination was normal and he had very soft right flank tenderness. Urine culture was negative.

**Figure 1:**
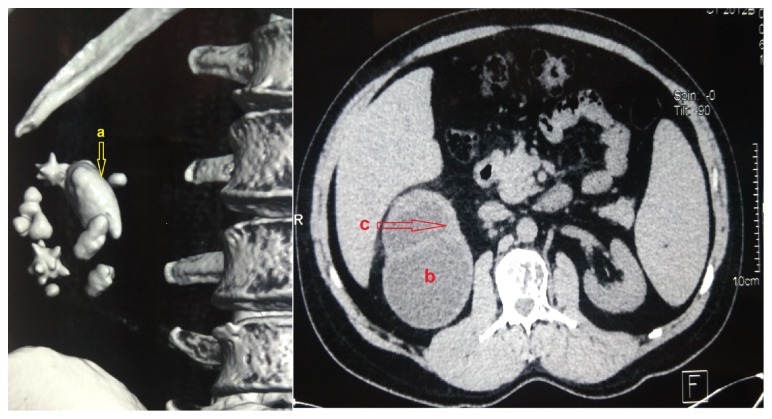
CT scan demonstrating large renal calculi and upper pole hydronephrosis: (a) large right renal pelvic calculi; (b) upper calix hydronephrosis; (c) thinned surrounding renal cortex.

Radiologic imaging including ultrasound and CT-scan showed a large right renal pelvic calculi and stones in the mid and inferior calix. It also showed upper calix hydronephrosis and thinned surrounding renal cortex (Figure 1). We proposed percutaneous nephrolithotomy (PCNL) but the patient refused any percutaneous or laparoscopic surgery. He underwent open surgical procedure. The upper pole of the kidney was adherent and surrounded by fibrotic tissue. We performed pyelolithotomy. While extracting the calculi, the urine was purulent so bacteriological samples have been taken. Pelvic suture was made after we placed Double-J ureteric stent.

Pus culture grew group D salmonella greater than 10^4^ colony-forming units (cfu) per mL of urine. Blood culture and HIV analysis were negative. Guided by measured antimicrobial susceptibility, we prescribed third generation cephalosporin (cefotaxim 2 g^*∗*^3/g IV) for 14 days. The patient made a steady recovery and 16 days after admission he was discharged home.

## 3. Discussion

Urinary tract infections due to NTS are uncommon. It was mentioned the first time in 1946 [3]. Its frequency oscillates between 0.015% and 0.118% [2, 4]. It is commonly seen in infants and patients older than 60 years, not in healthy adults like our patient [5]. In our case, UTI was caused by group D salmonella but in a study including 799 UTI, the most frequent serotypes were C1 and E [6]. NTS can infect the urinary tract either hematogenously or directly via the urethra especially in women [4]. Several predisposing factors are incriminated in NTS–UTI such as immune deficiency, chronic illness, immunosuppressive therapy, and abnormalities of urinary tract [7, 8]. Our patient had no chronic disease or immunosuppressive therapy, but urolithiasis predisposed him to NTS–UTI. In his study, Paterson et al. [9] found that only 3 patients from the 23 included had structural abnormalities of urinary tract and none of them had immunosuppressive disease. However, Tena et al. [8] found that all the 19 patients of the study had chronic disease or urologic abnormalities. In the literature, NTS–UTI do not differ clinically from UTI caused by other bacteria [2, 4, 8]. Our case is the first reported asymptomatic NTS pyonephrosis. Indeed, in other reported cases of NTS pyonephrosis, the patients was febrile and one of them developed a systemic inflammatory response syndrome (Table 1) [10, 11].

## 4. Conclusion

NTS urinary tract infection is rare. When diagnosed, it should lead to investigations looking for immunosuppressive illness or urological abnormalities including urolithiasis. NTS pyonephrosis can be life threatening but asymptomatic forms can be seen.

## Figures and Tables

**Table 1:** Characteristics of patients with pyonephrosis due to non typhoidal Salmonella.

Case no (ref)	Age (Y)/sex	Underlying disease(s)	Immuno suppressive therapy	Urologic predisposition	Salmonella serotype	Source of isolate(s)	Antecedent of diarrhea	Treatment	Outcome
1 [10]	47/M	None	None	Ureteral stone	Salmonella enterica	Urine and blood culture	No	Ceftriaxone gentamicin nephrectomy	Cured
2 [11]	7/M	None	None	Uretropelvic junction stenosis	Salmonella infanitis	Urine culture	No	Fosfomycin norfloxacin	Cured
Our patient	50/M	None	None	Renal stone	Group D salmonella	Urine culture	No	Pyelolitotomy cefotaxim	Cured
